# Postoperative wound infections after a proctectomy—Patient experiences

**DOI:** 10.3402/qhw.v11.30393

**Published:** 2016-02-18

**Authors:** Karin Hassel, Kristin Andersson, Inga-Lill Koinberg, Berith Wennström

**Affiliations:** 1 Department of Surgery, Skaraborg Hospital, Skövde, Sweden; 2 Institute of Health and Care Science, The Sahlgrenska Academy, University of Gothenburg, Gothenburg, Sweden; 3 Department of Anaesthesia, Skaraborg Hospital, Skövde, Sweden

**Keywords:** Nursing, postoperative infections, colorectal surgery, patient experiences, symptoms

## Abstract

Poor perineal wound healing and infections after proctectomy surgery cause a significant proportion of physical and psychological morbidities, such as pain, leakage, and abscesses. In the long run, some of these symptoms will lead to extended periods of hospitalization. These kinds of postoperative complications are also associated with delays in possible chemotherapy treatment. The aim of this study was to describe patient experiences of perineal wound infections following proctectomy due to rectal cancer, and the importance of the communication with and the self-care support from the nurse for these patients. Five women and five men (61–87 years, median age 71 years) were included and interviewed. A qualitative content analysis of the interviews was carried out and the following main categories emerged: “Managing postoperative complications,” “Being independent,” “Feeling safe,” and “Accepting the situation.” A perineal wound infection after a proctectomy is devastating for the individual patient. The limitations and changes to the patients’ lives turn into new daily routines, which force them to find new ways to live and to accept the situation. For many of them, the infections remained for several months and, sometimes, for years. The ability to lead an independent life is drastically reduced, but through continuity in care it is possible to create a feeling of safety. Information, communication, and self-care support are all important and valuable factors for recovery. Specialized care containing an action plan is therefore needed in clinical practice to reduce the number of perineal wound infections postoperatively and should be initiated when the patient is discharged from the ward and continue until recovery.

Colorectal cancer is the fourth most common type of cancer in Sweden, after prostate, breast, and skin cancer, and is more common among men than women. The median age for rectal cancer is 74 years (The Swedish Regional Cancer Centres, 2015). Surgery is the most important intervention to achieve cure and is often preceded by radiotherapy, sometimes in combination with chemotherapy.

Perineal wound complications after abdominoperineal resection (APR) is a common and significant problem, including infections and delayed wound healing, especially after neoadjuvant radiochemotherapy (Bullard, Trudell, Baxter, & Rothenberger, 2005; De Hass et al., 1995). These complications lead to prolonged hospital stays and long periods of wound care requiring home nursing or multiple visits to an outpatient facility. Severe cases may require vacuum therapy, so-called vacuum-assisted closure (VAC), to speed up wound healing. Patients with wound complications are also at greater risk of local relapses, as the adjuvant chemotherapy has to be postponed. In addition, chemotherapy has no effect after 12 weeks and cannot be administered during an ongoing infection (Schmoll et al., 2012). This, in turn, may influence long-term survival (Wiatrek, Thomas, & Papaconstantinou, 2008). Welsch et al. (2013) studied wound complications and quality of life in patients who had undergone APR. Eleven of the 30 patients (36.6%) included in the study developed perineal wound complications during their stay in hospital. Three additional patients were diagnosed with a perineal wound infection after discharge thereby increasing the wound complication rate to 46.6% (14/30). Symptoms that developed after discharge included pain, impotence, and faecal and urinary incontinence. The patients also experienced fatigue, sleep disturbances, and loss of appetite (Welsch et al., 2013). Delayed perineal wound healing may also involve a risk of persistent symptoms such as pain, paraesthesia, sitting disability, tension, tingling, and cramps (Asplund, Prytz, Bock, Haglind, & Angenete, 2015).

We performed a retrospective review of records at Skaraborg Hospital, Skövde, Sweden, resulting in a study describing the prevalence of postoperative perineal wound infections after proctectomy. The survey showed that 27 (66%) of 41 patients developed postoperative infections and delayed wound healing. The results have in part been presented at the National Swedish Congress for Surgeons (Hassel, Wennström, Sjöskog, & Skullman, 2013). Patients who have undergone proctectomy and develop a postoperative wound infection suffer troublesome symptoms. This impacts their well-being negatively, as the number of contacts with the health service increases and continued oncological therapy may be delayed (Tevis et al., 2013). To our best knowledge, there are no studies describing how patients experience their life situation when they have developed a perineal wound infection after a proctectomy. The aim of this study was to describe patient experiences of perineal wound infections following proctectomy due to rectal cancer, and the importance of the communication with and the self-care support from the nurse for these patients.

## Methodological approach

### Participants

The inclusion criteria specified Swedish-speaking patients who had developed a perineal wound infection following proctectomy, without consideration of age, education, or social status. Five women and five men (61–87 years, median age 71 years) were included. The patients had undergone proctectomy in 2010–2013. Two of the patients were working and the other eight were retired. Six of them were single and the other four were living with a partner.

### Data collection method

Two stoma therapists who were not associated with the study carried out the selection of patients. They reviewed the records for patients who fulfilled the inclusion criteria. The patients were thereafter contacted by telephone and asked if they would participate in the study. All the patients who were approached were willing to participate. A letter was then sent to their home address, with written information and a consent form that was later signed by the subjects and those responsible for the study in connection with the interview. The patients decided the time and place of the interview; three of them chose to be interviewed at home and seven at the hospital. Oral information about the study was given again just before the interviews started. All the interviews began with the question, “How did you experience the period after your operation when you developed a wound infection?” They were concluded with the questions, “Is there anything else you would like to tell me about?” and “Are there any other questions that you think I should ask you?” Follow-up questions were then asked about wound infections, information, and self-care support. The taped interviews were transcribed verbatim immediately after the interview by the two first authors. The interviews lasted between 30 and 90 min and were carried out for a week in the winter of 2014.

### Data analysis

A qualitative content analysis, described by Graneheim and Lundman (2004), was used during the analysis process. To grasp the messages in the data, this method required multiple interpretations and readings of the text to identify sequences that were condensed and further shortened into meaning units. Based on dissimilarities and similarities, the meaning units were thereafter translated into codes and further into sub- and main categories (see example in Table I).

**Table I:** Example of analysis process according to Graneheim and Lundman (2004).

Meaning units	Condensed meaning units	Code	Subcategory	Main category
I couldn't manage on my own like I wanted to. I was dependent on a great deal of help	Unable to manage by myself, dependent on help	Visit to hospital, healthcare centre, change of dressing	Being restricted. Being dependent on help	Being independent

### Ethical considerations

Written consent was obtained from the head of the clinic. Ethical considerations in the present study concerned written and oral-informed consent from the patient in accordance with the provisions of the Ethical Review Act (2003:460). The patients were informed about the voluntary nature of their participation and that they could withdraw at any time without stating a reason and without any implications for their future contacts with the health service. According to the Ethical Review Act, personal data must be treated confidentially so that no unauthorized persons are given access to the information and must not be disclosed for any other purpose than the study in question. All the patients were given a sequential code in the order that their interviews were made. The authors of this study have complied with the ethical guidelines mentioned above.

## Findings

The analysis of the data from the 10 interviews resulted in four main categories: “Managing postoperative complications,” “Being independent,” “Feeling safe,” and “Accepting the situation.” These main categories, together with subcategories, reflected the experience of living with a perineal wound infection following proctectomy and the importance of communication with and the self-care support from the nurse for these patients (Table II).

**Table II:** Overview of condensation according to Graneheim and Lundman (2004).

Main category	Subcategory
Managing postoperative complications	Managing early complications Living with long-lasting complications
Being independent	Being dependent on help
	Feeling isolated
	Being restricted
	Performing self-care
Feeling safe	Experiencing continuity
	Being seen
	Experiencing support
	Receiving information
Accepting the situation	Having an attitude The colostomy becomes a secondary problem

### Managing postoperative complications

A postoperative wound infection following proctectomy was a complication that involved both small and large changes to the patient's life situation. The main category, “Managing postoperative complications,” described the patients’ various physical symptoms, such as leakage, smarting, pain, and unpleasant odour; however, the patients also experienced psychological symptoms, such as fatigue and depressed mood. All the patients had a prolonged wound-healing process, lasting from 18 months to 3 years. For one of the patients, the wound was still unhealed after 3 years. The majority of the patients who were interviewed developed a wound infection within the first few days after the operation when they were still in the hospital ward. This main category was divided into the subcategories of “Managing early complications” and “Living with long-lasting complications.”

#### Managing early complications

The postoperative wound infection started in different ways in the 10 participants who were interviewed. One patient fell on the floor in connection with postoperative mobilization, the wound split open, and an infection developed. Another patient described the appearance of a smell during the time at the hospital. The majority of patients described and unpleasant odour which made them feel uncomfortable together with other people. One patient developed an infection at home.

I was just lying there and noticed a bad smell; it had to be infected, I thought, and I don't know how long it took, but I think it must have been 4–5 days before they opened it and looked at it. (Patient 5)

All the patients described leakage and a smarting pain from the wound. Some of the patients described more intense pain. The first period with an infection was described as “difficult” and “troublesome,” and fatigue was a common problem.

#### Living with long-lasting complications

The majority of the patients had continued problems with leakage and smarting pain during the entire prolonged wound-healing process and sitting was difficult. All the patients also needed frequent changes of dressings. Two of the participants described severe pain. In connection with changing of dressings at the hospital, they were given opioid drugs as prophylactic analgesics and certain extensive changes of dressings, and VAC had to be carried out in surgery under general anaesthesia. The continued process with changing of dressings was managed at the healthcare centre, through the home-nursing service or by a close family member.

It was really difficult during the first two years. Still, today, after 3.5 years, the skin feels tight—as if there wasn't enough of it—when I'm working and exert myself. (Patient 2)

Half of the patients were treated on one or several occasions with VAC to speed up wound healing. They continued to experience symptoms from their wound infection also after the wound had healed. They could not sit without an aid in the form of a seat cushion and experienced tenderness and pain during strain and heavy lifting.

### Being independent

The main category of “Being independent” involved restrictions of the patients’ autonomy, as all the patients in the study became dependent on help in one way or another in order to manage the wound. The ability to manage on their own emerged as an important aspect for a majority of the patients. This category was divided into the subcategories of “Being dependent on help,” “Feeling isolated,” “Being restricted,” and “Performing self-care.”

#### Being dependent on help

Because of the location of the wound, it was difficult for the patients to change dressings themselves, which led to a need for help. The majority of the patients mentioned in the interview that they had never seen the wound. The patients who were treated with VAC experienced an even greater dependence on help during the changing of dressings.

I couldn't manage on my own like I wanted to; you become dependent on quite a lot of help. (Patient 1)

All the patients stated in the interviews that they had scheduled appointments at the healthcare centre in the mornings and sometimes in the afternoon. In some cases, they also had visits from the home-nursing team at home in the evening because of the need for frequent changes of dressings. This resulted in a very tight schedule and the need to use special transport to a healthcare facility, or some other form of transport, often provided by a family member. The patients vacillated between a feeling of gratitude for the help with something they could not manage themselves, and the discomfort of having many different individuals visiting their homes.

#### Feeling isolated

Troublesome symptoms from the wound infection and the need for frequent changes of dressings, scheduled appointments at hospitals and healthcare centres, or visits from the home-nursing team, affected the patients’ social life. They described how all these factors taken together prevented them from leaving home in some cases. Three of the patients were hospitalized for long periods during their wound infection and this led to a feeling of being cut off from home, friends, and family.

The worst thing was the smell. It was awful! The last couple of times I travelled with the bus, it was no fun sitting there among people with the pump (VAC) and being smelly. It was no fun at all. At home, I was lying down because I couldn't sit up to do things and I had to lie on the couch and eat my meals. (Patient 8)

#### Being restricted

All the 10 patients reported that their lives had been restricted in one or several ways. For the five patients who were treated with VAC, life between dressing changes was even more restricted, as they had to keep the pump with them at all times. At the same time, it helped in speeding up the wound healing.

Of course, it was rather difficult to begin with, and then there was that device—it was a bit awkward to carry it around to begin with, but I must say that it was exceptionally good; it helped me and I healed faster. It was a bit of a job to carry it around, but other than that, it wasn't difficult, but I didn't really want to go out with it. (Patient 1)

As the majority of the patients had problems with sitting and were dependent on frequent dressing changes due to leakage, they had to plan their lives accordingly. For those who worked, aids in the form of seat cushions and proximity to a toilet were absolute musts. Their social lives were restricted, and meeting friends was often difficult; for some of the patients, the changing of dressings was all they had time for and managed to handle in a day.

#### Performing self-care

To some extent, all the patients in the study performed self-care on some occasion during the course of their wound infection. Because of the constant leakage, the bandages or compresses sometimes fell off before the home-nursing team arrived or before the scheduled visit to the healthcare centre. The patients then had to change the dressings or bandages themselves to the best of their ability. Four of the participants received self-care support from a nurse, to be able to handle the dressing changes themselves at home. These patients felt less controlled and restricted in their social life and felt that they were responsible for their own situation.

The health care centre checked once a fortnight. Or my wife came along and was told how to change the dressing. When we had been informed, we were given material to change the dressings ourselves. It was valuable, at the same time as it was a huge responsibility. (Patient 7)

### Feeling safe

The main category of “Feeling safe” includes several components that contribute to a feeling of safety in the patient and was divided into four subcategories: “Experiencing continuity,” “Being seen,” “Experiencing support,” and “Receiving information.” The patients had to hand themselves over to other people and show them their private parts. They talked about being met with respect. Perceiving that their integrity was maintained was important to induce a sense of safety. Support and follow-up by the nurse were appreciated, and another important aspect in this category was being recognized as an individual.

#### Experiencing continuity

In this study, continuity meant that the majority of the patients expressed a wish to meet the same nurse at their visits to the hospital and the healthcare centre. Even though the patients experienced good treatment by the home-nursing staff, the large number of new people passing through their home made them feel less safe.

It was fine to go to the health care centre where I met the same nurse each time. The toughest part was the visits by the home-nursing team with different people all the time. It is, after all, rather intimate business, so I thought that was the most difficult bit. (Patient 3)

#### Being seen

Because of the location of the wound, the patient had to undress and show his or her intimate parts every time the dressing was changed. Meeting the same healthcare personnel and the same nurse inspired confidence and trust in the patients, which facilitated the dressing situation. It was also obvious that it was very important for the patient to be recognized, that the staff saw the individual behind the patient.

After all, when you take off your clothes and turn your bottom up, you feel a bit silly. But not there—they were all really nice. (Patient 4)

#### Experiencing support

All the patients were satisfied with the stoma clinic at the hospital. They felt that they received good support and follow-up from the nurses at the clinic. Accessibility and the level of knowledge were also perceived as high and the patients felt that they could always turn to the clinic; that the door “was always open.” The patients were satisfied with the treatment at the healthcare centre, but emphasized that it is important to be given continued access to the stoma clinic, as the staff there has expert knowledge about their situation.

I got support from the stoma therapist who knows what she's talking about, and I felt that she is closest to the patient. They say that they have to refer the patient after a number of days to the healthcare centre, and the healthcare centre says that they haven't got the time to be experts at this. Now, if you've been through such a major change to your body, like a colostomy, the health service should allow you to come back for check-ups every now and then. (Patient 7)

#### Receiving information

A majority of the patients reported that they had received inadequate information about what is to be considered normal after a proctectomy. Some of the patients who developed a wound infection at home after discharge from the hospital had missed the signs of an infection and understood leakage as being a part of the normal process. The interviews also reflected the opinion that the information concerning the colostomy and the stoma care had been satisfying. Some patients with an ongoing wound infection who were discharged from the ward lacked information about expected symptoms and the duration of the healing process. It was not until they came back to the stoma clinic that they felt that they were given support and information.

There was no talk of pain after the operation, we didn't have a clue that it would last this long. I've no idea whether this was due to ignorance or because they didn't care or didn't have the time. I hope that future patients will get some backup information about how tight the skin may get and that you can get all those problems. No one said a word about it! (Patient 2)

### Accepting the situation

Common to all the patients was the need to learn to live with the complications, which, for many patients, lasted for several months and, sometimes, for years. They accepted the situation and endured it, some of them because they were grateful to be alive, as they had recently lost a close relation. Others mentioned their attitude to life and what they were like as people. The main category of “Accepting the situation” was divided into two subcategories: “Having an attitude” and “The colostomy becomes a secondary problem.”

#### The colostomy becomes a secondary problem

A majority of the patients reported that their greatest fear before the operation was to get a colostomy; how to manage to live with a colostomy and take care of it single-handedly. However, after they developed a wound infection, with all the resulting problems, the colostomy became a secondary problem that was easy to manage compared with the perineal wound.

I was mainly worried about how to manage the colostomy after leaving the hospital. But they showed me what to do and it worked just fine. It was no trouble. The colostomy itself wasn't that difficult, but the wound infection was really troublesome. (Patient 4)

*Having an attitude*. Most of the patients mentioned at some point during the interview that their way of being, their personality, had helped them accept the situation. One patient described how his upbringing had given him good self-confidence and good self-esteem. This had been very valuable later in life in order to deal with problems. The patients described an attitude of a “fighting spirit” and that they had no other choice.

Patience is the only thing you need, otherwise it won't work. But I am grateful that it worked as it did. These four years have just disappeared, but I am grateful that I got another four years. My daughter only got four months after her diagnosis. (Patient 5)

## Methodological considerations

When determining the inclusion criteria, no consideration was given to age, social status, or education. The results may have been influenced by the age of the patients (61–87 years). The results may also have been influenced by the fact that the authors work with this group of patients and meet them at different stages of their illness; a professional pre-understanding. On the other hand, these previous insights may have increased the trustworthiness of the study because the knowledge about the diagnosis and the illness facilitated the condensation and analysis of the material. According to Malterud (2001), previous insights are the knowledge that we bring with us to a study. This knowledge may be a strength in the project but may also be a burden. It is important to ensure that the previous insights do not outweigh the empirical material that is gained from the study. Hence, it is not “wrong” to have previous insights about the area of study, as long as the researcher is aware of this and considers it while analysing (Malterud, 2001). To achieve trustworthiness, different steps of the analysis have been reviewed and discussed on the basis of the concepts of credibility, dependability, and transferability (Graneheim & Lundman, 2004; Lincon & Guba, 1985). During the course of the process, we ensured that the analysis corresponded to the aim and that the whole material was analysed at the same time, both separately by each author, and by all the authors together. The context and the participants are also described as clearly as possible to facilitate the transferability of the results.

## Discussion

This study showed that a perineal wound infection following proctectomy is devastating for the individual patient. The patients described problems with leakage, smarting, and constant pain of varying intensity and degree. All the patients experienced these problems, regardless of the duration of the wound-healing process. However, patients whose wound-healing process lasted more than a year (three patients) described the pain as being more pronounced, and they also emphasized the unpleasant odour, resulting in fatigue and depression. The patients described problems with sitting because of the location of the wound, and all of them had experienced difficult and painful dressing procedures at more or less frequent intervals, requiring prophylactic analgesia. Their quality of life was negatively impacted. All the patients were dependent on help to manage the changing of dressings because the wound was located at a site that was difficult to reach. They felt that they were prevented from leading a normal social life, which led to isolation of varying degrees. They also described how the large number of scheduled appointments in order to have the wound attended to severely controlled their lives.

A study by Mudge, Meaume, Woo, Sibbald, and Price (2008) investigated patient experiences of wound-related pain, where patients from three different countries were interviewed in focus groups. The patients in the Mudge et al. study reported problems similar to those reported in our study. They associated the changing of dressings with pain and prophylactic analgesia was required before treatment. Like Hollinworth (2005), we therefore argue that it is necessary for the nurses to be updated about which dressing material is the most suitable for the individual patient as the appropriate dressing material can prevent pain, unpleasant odour, and leakage. It is also important to learn from the problems described by the participants in our study, in order to prevent pain with the right pharmacological treatment at the right time.

This is in line with Chen et al. (2013) who described the effectiveness of a wound-healing programme with the focus on self-care. The patients in the experimental group received wound care and dressing instructions, involving both theory and practice. The control group received care according to the procedures in place at the hospital in question, with limited information compared with the amount of information given to the experimental group. The results showed that the experimental group experienced greater satisfaction and there was a significant difference in the degree of wound healing, with the wounds in the group receiving wound care and self-care support healing faster. The wound care and the self-care support resulted in benefits, both for the patients and their family members since they gained knowledge to create the best possible opportunities for rapid wound healing. Although this involved a major responsibility for family members who helped the patient at home, it was also seen as valuable because they gained insights and knowledge about the wound-healing process.

Theofanidis (2006) claim that patients worried the most when they received inadequate information, and that surgical patients were exposed to greater stress than medical patients because of the greater worries linked to undergoing a surgical intervention. The author also argues that stressed and worried patients have a longer postoperative recovery period and may experience more pain with a higher incidence of wound infections, which, in turn, leads to longer periods of hospitalization (Theofanidis, 2006). In addition to the illness itself, receiving a diagnosis, undergoing surgery, and developing wound complications involve a large number of contacts with the health service. The patient is placed in a situation of dependence.

Most of the patients in our study reported that they were not sure what the postsurgery situation was supposed to be like; perhaps leakage was perfectly normal? They were disappointed with the inadequate information about the perineal wound-healing complications that may develop *after* a proctectomy. Park et al. (2014), studied patient expectations on bowel function and how this would affect their life after rectal cancer surgery. The results showed that information, but also personal attitude, was important. However, despite adequate information and a positive attitude, there was great uncertainty and concern about their bowels function and how this would affect their lives in the future. The nurse plays, thus, an important role to facilitate for the patient pre- and postoperatively, to communicate and listen to the patient's need, and to act as a coordinator between the different services that the patient is being shuttled between.

To live with a perineal wound infection was a difficult situation in many different ways. The patients emphasized that the only option was to accept and make the best of the situation, and that their personality and attitude had helped them. Hence, to accept the situation does not mean that the patients accepted their wound infection, but that they were forced to deal with the outcome of it. These findings show the importance of supporting patients, helping them improve their self-esteem, and creating a positive attitude in the individual patient, as such an attitude and a “fighting spirit” helped a majority of the participants through this difficult time. However, all the participants in our study were satisfied with the continuity afforded by meeting the same nurse at all visits and receiving support and information, which they experienced as greatly satisfying and valuable, although two of the patients pointed out that they had not received adequate information on a number of occasions, which made them feel exposed and lonely.

Carlsson, Berntsson, Hallen, Lindholm, and Persson (2010), investigated patients’ quality of life prior to surgery and during the recovery period after rectal cancer and a colostomy, where one of the fears expressed by the patients was related to receiving a colostomy. They were also worried about dying early, loss of bowel control, and being a burden or dependent on others. The quality of life among these patients was significantly impaired preoperatively, and this finding also applies to the patients interviewed in our study. Several studies highlight problems experienced by patients living with a stoma, where symptoms such as leakage, unpleasant odour, pain, skin problems at the stoma site, hernia, depression, and anxiety have been described (Bullen et al., 2012; Feddern, Emmertsen, & Laurberg, 2015). Symptoms can occur alone from another, but more often, multiple symptoms are experienced simultaneously. If two or more symptoms occur at the same time, they are likely to affect each other, for example, pain is worse when fatigue or nausea occurs at the same time and the intensity of a symptom, such as pain, may “dominate” or “mask” other symptoms, for example, fatigue and depression (Dodd et al., 2001). In our study, Dodd et al.'s (2001) evidence-based statement might explain why the patient's fear of living with a colostomy became secondary in relation to the postoperative perineal wound infection. Despite problems with the stoma, the patients experienced the colostomy as the “easy part” to manage.

However, we concentrated on the patients experiences of symptoms, ill- and well-being, and on finding ways to improve our current strategies. The findings of the interviews reflect the major impact of developing a postoperative perineal wound infection on the patients’ quality of life compared with the patients who follow a normal wound-healing process.

## Conclusion and clinical implications

A perineal wound infection following proctectomy is devastating for the individual patient. The nurse must therefore work preventively to reduce the risks of a postoperative wound infection. A new programme of measures has begun to take shape to reduce the number of postoperative wound infections for patients who need specialized care. This programme should be initiated at the time of discharge from the ward and continue until full recovery and include continued development work with a contact person/ stoma therapist. All patients who develop a wound infection should also receive self-care instruction and support, together with their family members, including education in anatomy, physiology, and wound care.

The results show a high degree of concordance among the patients when they suffer complications, not only during the acute phase of the infection but also in the case of long-lasting complications that remain after wound healing and, for some patients, persist for several years. This leads to an increasing number of contacts with the health service and may delay continued oncological treatment. It also involves increased health economy expenses, including prolonged care periods, readmission to hospital, and repeated visits to and treatments by district and municipal care healthcare facilities.

Finally, in order to promote self-care support, the very important findings concerning the patient experiences of “Feeling isolated” and “Being restricted” should be explored in future research.
